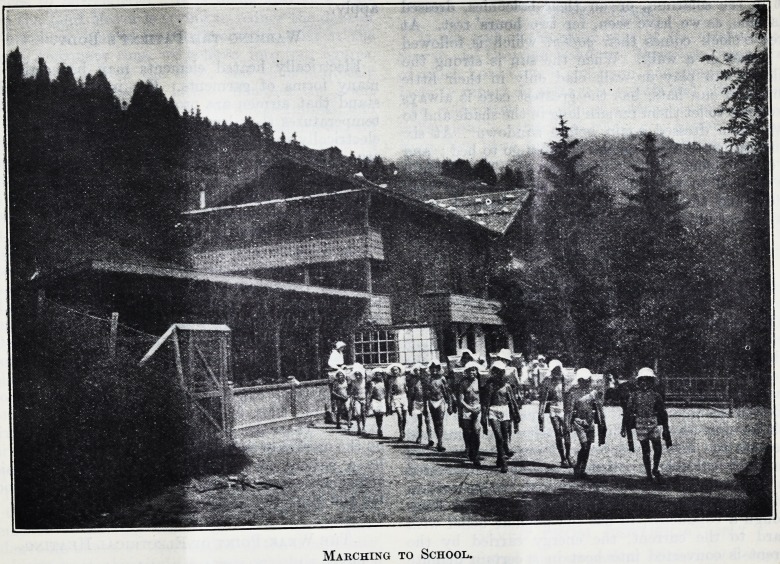# The School in the Sun

**Published:** 1924-11

**Authors:** 


### THE SCHOOL IN THE SUN.

### A DAY AT LES NOISETIERS.

For years I had heard of the "School in the Sun" as a sort of Mecca for delicate children, and when I came to Leysin the more I learned of Dr. Rollier's methods the more eager I was to see Les Noisetiers. At last the opportunity occurred, and on a perfect summer afternoon we set out, a party of four enthusiasts, and motored along the Sepey Road. In a few moments we were out of sight of Leysin with its endless clinics and its 2,500 invalids, and found ourselves in the valley of the Grande Eau, rejoicing in its verdant pastures, shady pine woods and picturesque Swiss dwellings. Alighting at the gate of a chalet larger than the rest, we were heartily welcomed by the *directrice* of Les Noisetiers. We had just time to make the tour of the house, which after all is not unlike an English school in its internal arrangements, when the children emerged from their two hours' "silence" in the solarium, and flocked into the dressing-rooms to tidy themselves for their afternoon meal. We then proceeded to tea under shady trees, and soon had an opportunity of taking stock of the healthy, happy appearance of the children, who partook of their hot chocolate and bread quite close to us in front of the house.

Their *goûter* over, our young performers retired to undress, and soon reappeared clad only in little white pants and shady white cotton hats. With their desks slung on their backs they came forward in Indian file singing lustily. After going through a series of marching exercises they formed up in rows, unfolded their desks, and sat down as though ready for lessons. Such activity always precedes class work. Occasionally the children armed with their desks go further afield and seek in their own special wood a secluded spot in which to work. In winter, when the sun is strong, the more skilled among them make similar excursions on skis, but such feats can only be achieved by those who have been long at Les Noisetiers, and have thus become thoroughly acclimatised. The desk drill ended, we were led up to an ash playground, about the size of a tennis court, behind the school, whither we were followed by a row of merry, brown little people, now armed with Swiss flags. After some preliminary marching, accompanied by a spirited song about the *cher drapeau*, our young athletes formed up and gave us a further display of their prowess. What struck us most was the obvious joy of unfettered movement, and the ease with which the little chests expanded. The teacher seemed to enjoy herself as much as the children, and it was with regret that we saw them file off once more, this time to amuse themselves with games, jumping and skipping on the playground in front of the house.

TEACHER'S LITTLE JOKE.

Meantime our guide led us back to our seats under the trees, whence she called some of the children to speak to us. One had lain for years in plaster of Paris (not at Leysin, where it has long since been discarded) with a tuberculous spine; another had been a martyr to bronchitis; another had been so thin on arrival that he looked like a living skeleton; another had been disfigured by swollen glands, which had resumed their normal size under the kindly influence of the sun; while yet another, whose father had died of phthisis, had herself been threatened with lung trouble. All of these forty-two children, it appears, had that in their history which would cause grave anxiety regarding their health were they required to live under ordinary conditions, and as we watched them at play we began to realise that each one of them had been saved from crippledom, a sick-room, or a grave!

Thus it was with redoubled interest that we plied our hostess with questions when the children, who are not allowed to remain long in the shade, had one by one returned to their play. Let us try to condense the information so freely accorded to us. After seven years of experience of the sun cure, Dr. Rollier fulfilled a long cherished ambition in 1910 when he inaugurated "The School in the Sun" for delicate children. At first Les Noisetiers contained only ten children, with merely a housekeeper and one teacher to look after them, but within a year there were twenty pupils and a proportionately enlarged staff. Now the chalet has been improved and extended, and the playground and solarium have been added. The present staff consists of the original housekeeper, a *directrice*, who is also a trained nurse, a certificated school mistress, and three kindergarten teachers who undertake not only to teach gymnastics and supervise the children's play and outings, but to mend their clothes and see that they are tidy in their persons, rooms and cupboards.

The work of these devoted women is no sinecure, and would probably turn the hair of English school mistresses grey! Not only do the children range in age from four and a half to thirteen, but they belong to eight or ten different nationalities, and speak almost as many languages. To crown all, besides the varieties of creed to which we are accustomed at home, it is literally true that Les Noisetiers has harboured "Jews, Turks and Infidels." How, we ask aghast, can health be acquired, discipline established, and a high moral tone maintained in a school containing such extraordinarily diverse material? It would seem that the impossible is accomplished by means of personality and method. The former, of course, we cannot explain; the latter our guide sketched for us. Children are admitted to the school as vacancies occur, so that there can be no terms and sessions. There are no regular holidays, except the great fête days, but individual children are exempted from lessons if they seem to require a break. All pupils are examined by the doctor before entering Les Noisetiers, and are seen by him from time to time, but illness is almost unheard of among the children, and the few colds that occur can usually be cured by the prompt methods adopted by the staff.

MARCHING TO SCHOOL.

The daily programme is as follows. The children rise at 6.30, and breakfast at 7. From 7.30 to 9.30 they do their lessons, in the open air when the weather permits. Immediately thereafter follow gymnastics, especially breathing, exercises. From 10 to 12 comes the actual "cure," when the sun shines; otherwise the time is occupied either by a walk or by games according to the exigencies of the weather, variable even here. The sun cure, which might almost be called the *raison d'être* of the school, is not so simple as one might imagine, and must be administered gradually. There are pitfalls for the unwary, but these have been avoided by the skilled workers at Les Noisetiers; the children tan rapidly, and are soon sufficiently pigmented to benefit from the fierce rays of the mountain sun. Dinner follows at noon, and thereafter the little ones lie out in the solarium, or on their balconies, dressed this time, as we have seen, for two hours' rest. At three o'clock comes their *goûter*, which is followed by games or a walk. When the sun is strong the children can play or walk clad only in their little pants and sun hats, but the greatest care is always taken not to let them remain long in the shade and to have them dressed again before sundown. At six comes supper; at seven the children go to bed; and by eight o'clock all light must be out. The children are trained to wash themselves all over with cold water, and they have each two hot baths a week —a liberal allowance for Switzerland!

## Figures and Tables

**Figure f1:**
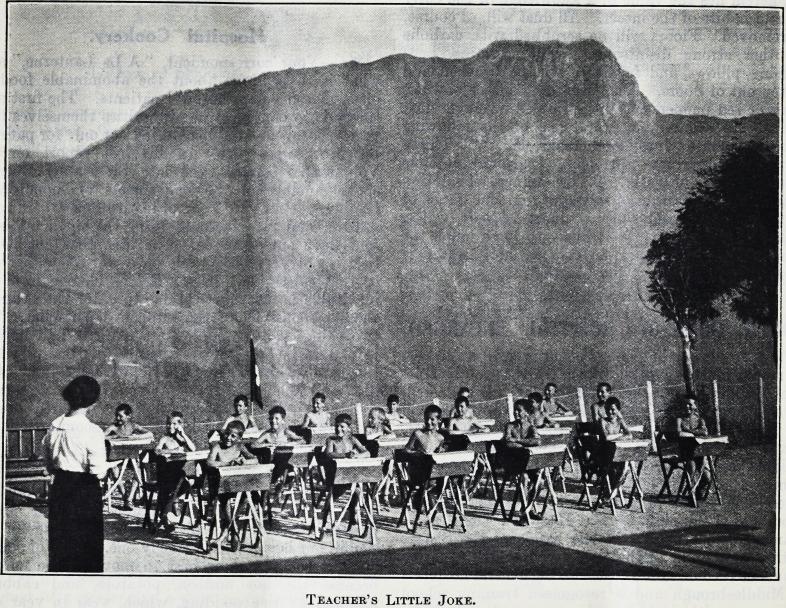


**Figure f2:**